# Conditions of Membership in the Ninth International Medical Congress

**Published:** 1887-08

**Authors:** 


### CONDITIONS OF MEMBERSHIP IN THE NINTH INTERNATIONAL MEDICAL CONGRESS.

"Rule 1. The Congress will consist of such members of the regular medical profession as shall have registered and taken out their ticket of admission, and of such other scientific men as the Executive Committee of the Congress shall deem desirable to admit. The dues of membership for residents of the United States will be ten dollars ($10). There will be no dues for members residing in other countries. Each member will be entitled to receive a copy of the *Transactions* of the Congress when published by the Executive Committee.

This rule, plainly defining the conditions for acquiring membership and participation in the approaching International Medical Congress, was adopted and published in English, French and German,in Circular No. 1, issued and widely distributed in both this country and Europe by the Executive Committee more than two years ago. It was repeated in Circular No. 2, issued in July, 1886, and also republished in most of the medical periodicals of this country. And yet we notice that some of our State and local medical societies have appointed *delegates* to the Congress, and we are often receiving letters making inquiries touching the same subject. Hence we have again quoted the rule, and wish to state explicitly that the doors of the International Medical Congress will be thrown open to all members of the regular medical profession, in all countries where such profession exists, who may apply to the Registration Committee in Washington, D. C., enter their names in full on the roll, and takes their tickets of admission. Those residing in this country must pay at the time of registering the $10. From those residing in other countries no fee will be required.

In regard to the registration of educated dentists, about which there has been some question, it is sufficient to say that the same rule will be followed as governed at the

London Congress of 1881. The establishment of a Section of Oral and Dental Surgery. is a full admission that it constitutes a part of the domain of general medicine and surgery, and that all who, by education and proper legal authority, practice in that special department, are "members of the regular medical profession." At the London Congress they registered with the common prefix "Dr." as did a large proportion of eminent members of the profession in other departments. At the Congress in Washington it will be proper for them to register with the title Dr., M. D., D. M. D., or D. D. S., according to the terms of the authority conferring upon them the right to practice their profession.—*Journal of American Medical Asso.*

---

